# Conditional TransGAN-Based Data Augmentation for PCB Electronic Component Inspection

**DOI:** 10.1155/2023/2024237

**Published:** 2023-01-10

**Authors:** Chenglong Wang, Guanghan Huang, Zhiyuan Huang, Weiming He

**Affiliations:** ^1^School of Electronic Information and Electrical Engineering, Huizhou University, Huizhou 516007, Guangdong, China; ^2^Guangdong Provincial Key Laboratory of Electronic Functional Materials and Devices, Huizhou University, Huizhou 516001, Guangdong, China

## Abstract

Automatic recognition and positioning of electronic components on PCBs can enhance quality inspection efficiency for electronic products during manufacturing. Efficient PCB inspection requires identification and classification of PCB components as well as defects for better quality assurance. The small size of the electronic component and PCB defect targets means that there are fewer feature areas for the neural network to detect, and the complex grain backgrounds of both datasets can cause significant interference, making the target detection task challenging. Meanwhile, the detection performance of deep learning models is significantly impacted due to the lack of samples. In this paper, we propose conditional TransGAN (cTransGAN), a generative model for data augmentation, which enhances the quantity and diversity of the original training set and further improves the accuracy of PCB electronic component recognition. The design of cTransGAN brings together the merits of both conditional GAN and TransGAN, allowing a trained model to generate high-quality synthetic images conditioned on the class embeddings. To validate the proposed method, we conduct extensive experiments on two datasets, including a self-developed dataset for PCB component detection and an existing dataset for PCB defect detection. Also, we have evaluated three existing object detection algorithms, including Faster R-CNN ResNet101, YOLO V3 DarkNet-53, and SCNet ResNet101, and each is validated under four experimental settings to form an ablation study. Results demonstrate that the proposed cTransGAN can effectively enhance the quality and diversity of the training set, leading to superior performance on both tasks. We have open-sourced the project to facilitate further studies.

## 1. Introduction

A printed circuit board (PCB) is the carrier of many electronic components in electronic products. Electronic components must be assembled according to categories and in the right positions during the manufacturing of electronic products. In practice, the recognition and positioning of electronic components on PCBs has been the key technology in the manufacturing and assembly of electronic products. There have been mainly three types of recognition methods for printed circuit board (PCB) electronic components: traditional artificial visual detection methods, machine vision-based detection methods using image processing, and deep learning-based detection methods. Some other novel detection methods have also been developed, for example, automated x-ray detection and laser detection systems. However, they all have the shortcomings of high cost and failure rate, as well as slow detection. Traditional object detection techniques basically consist of three steps: first, identify the candidate region by using a sliding search window with different scales, next, retrieve the visual features of the candidate region, such as Haar features or HOG features, and lastly, classify the regions. There are inherent shortcomings in traditional object detection methods: (1) using sliding windows for region identification and selection has the problem of weak pertinence, high time complexity, and lots of redundant windows; (2) manually designed features are subjective and rely on an individual's prior knowledge, and the process of detection is cumbersome; (3) the methods are time-consuming, which cannot meet the needs of real-time detection.

In recent years, the automatic optical inspection technology (AOI) [1] has been used to detect PCB defects during its manufacturing. Compared with traditional manual inspection, it has multiple advantages, such as fast detection, low cost, and high accuracy. During the past decade's evolution of AOI technology, there are mainly three categories of methods: reference comparison, nonreference comparison, and mixing. The reference comparison method involves matching a given image based on a given standard target sample image, finding regions in the given image that have a high correlation with the target sample image and then using an algorithm to align the edge contours as closely as possible to achieve the target frame. The main challenge of this method is to align the reference image and the test image precisely, which requires a complex configuration process. Meanwhile, light and noise greatly impact the detection process, which can easily cause false alarms [2].

PCB electronic component identification tasks mainly aim to detect different types of capacitors, optocouplers, and diodes. Traditional artificial visual inspection methods and image processing-based machine vision detection methods have problems such as low accuracy, poor generalization ability, poor robustness, and lacking compatibility with multiple PCB electronic components. Eventually, they cannot meet the needs of manufacturing. Deep learning-based object detection methods have become the mainstream in the field. Deep learning methods demonstrate an advantage in automatic feature extraction. Typical object detection algorithms based on two-stage model of region detection classification include R-CNN [3], SPP-Net [4], Fast R-CNN [3], and Faster R-CNN [5]. Typical object detection algorithms based on regression single-stage models include SSD [6], RetinaNet [7], and YOLO [8]. The main challenges and difficulties of the identification of printed circuit board (PCB) electronic components are as follows: first, there are many types of PCBs and different design rules on the market; second, electronic components on the PCB and its features are complex and diverse; third, the PCB electronic component industry lacks a large number of samples of different types, resulting in data imbalance in traditional methods. Therefore, it is practically significant to design a method to expand the samples and increase the accuracy of the DNN prediction model.

In this paper, we propose a deep-learning pipeline for PCB electronic component inspection. The core technical contribution is conditional TransGAN (cTransGAN) utilized for data augmentation. The proposed cTransGAN, after extensive training, can generate high-quality synthetic PCB components and defect samples used to enhance the quantity and diversity of the original training set, leading to impressive performance gains in mean average precision (mAP). cTransGAN is featured with a TransGAN generator and discriminator, both conditioned on the class embeddings, which are latent representations of the classes in the dataset. These embeddings, served as inputs, can effectively guide the generator to produce an image belonging to a desired class; meanwhile, the discriminator is also guided to better distinguish real and generated images given the desired class.

To validate the proposed method, we conduct extensive experiments on two datasets, including a self-developed dataset for PCB component detection and an existing dataset for PCB defect detection. Also, we have evaluated three existing object detection algorithms, including Faster R-CNN ResNet101, YOLO V3 DarkNet53, and SCNet ResNet101, and each is validated under four experimental settings to form an ablation study. Results demonstrate that the proposed cTransGAN can effectively enhance the quality and diversity of the training set, leading to superior performance on both tasks. The code of this project is available at https://github.com/long-deep/pcb-detect.

The rest of this paper is organized as follows.  Section 2 reviews research work related to PCB object detection and data augmentation.  Section 3 explains our proposed model and dataset. In Section 4, several comprehensive experiments are conducted to evaluate the effectiveness of the proposed model. Finally, in Section 5, we conclude the paper and provide future work.

## 2. Related Work

### 2.1. Object Detection and PCB Electronic

In recent years, computer vision has made significant progress in object detection [9, 10], which has advanced the development of autonomous vehicles [11], robotics [12], and many other practical applications. The networks have achieved reliable performance, with stable, easy-to-use, open-source implementations [13] published. These implementations are also well documented, which is convenient for researchers to fine-tune their pretrained models for specific tasks. However, almost all object detection networks need to be trained on large-scale datasets to obtain good performance. Unfortunately, for the PCB component detection tasks, it is expensive to build a large-scale dataset to fine-tune such detection networks. In addition, due to intraclass variance, there is inherent ambiguity in classifying components. Therefore, we have studied methods that utilize the inherent structure in the data (that is, within the PCB board), which cannot be achieved by traditional detection methods.

As well known, there are different categories of electronic components with different shapes. CNN simulates the brain's visual cognition principles. Through dimensionality reduction, CNN retains the characteristics of the object even if the object reappears in a different scale, direction, and position. Therefore, CNN can be applied for the detection of electronic components. In [14], a novel graphical network block is proposed to refine the component features on each PCB. It can reach a 65.3% mAP of electronic component detection on the testing PCBs [14]. In [8], an improved YOLOv3 algorithm is proposed, adding an output layer that is sensitive to small objects. The paper also verifies the effectiveness of the algorithm in both real PCB pictures and virtual PCB pictures, which include many PCB electronic components. In [15], a fast object recognition method is proposed, which combines YOLO-V3 and Mobilenet. They use MobileNet to replace Darknet53, the original architecture in YOLOv3, to achieve lightweight and fast speed. However, a common issue in the above CNN-based electronic component detection methods is that the dataset is limited, which cannot enable CNN to learn PCB electronic components well, resulting in low accuracy of PCB component recognition. In this research, we build our dataset, including images of the same type of electronic components in four ways (up, down, left, and right), which provides the model with more accurate recognition data.

### 2.2. Data Augmentation Techniques

In industrial applications, the prediction accuracy of a deep learning model mainly relies on the size and quality of the training samples. The collection of samples for electronic component recognition takes a long time and is even difficult to obtain. The generative adversarial network (GAN) [16], as a generative model, can generate new synthetic instances of data that follow rather similar, if not exactly the same, distribution of real samples through continuous confrontation between the generator and the discriminator. At present, GAN has been widely used in different areas including image generation, style transfer, and many other fields [17–19]. Due to the limited size of the training dataset and the ambiguity of unknown electronic components, identification of unknown electronic components is still a challenging task. Deep learning-based image recognition usually requires lots of sample images for training. Data augmentation techniques should be adopted when there are limited images available for training. Based on this consideration, Abayomi-Alli et al. proposed an image augmentation technology [20] based on the random permutation of coefficients of within-class principal components obtained after applying principal component analysis (PCA). After reconstruction, the newly generated replacement image is used to train the deep network. Experimental results show that the method can improve classification accuracy and classification ambiguity in applications [20]. In the case of a small dataset, data augmentation has always been an effective method to reduce overfitting. Even though there are already a variety of augmentation techniques, such as horizontal flip, random crop, and Mixup, they are not suitable for object detection tasks because of the lack of labeled bounding boxes information for corresponding generated images. To address this issue, in [21], an unsupervised data augmentation framework using GAN is proposed. The authors proposed a two-step pipeline based on YOLOv4, which enables the generation of an image with the object lying in a certain position.

Recent advances attempt to explore the potential of generative models for image augmentation to address the issue of low training resources that has been a long-lasting challenge to train a deep learning model with satisfying generalization ability [22]. Compared to traditional augmentation methods, mostly based on image processing techniques, generative models such as GANs can capture the semantic features of images used for training and generate similar but different images to enhance the quantity and diversity of training data. Such capability of GANs has driven its usage in image augmentation for various computer vision tasks, including classification [23, 24], object detection [25, 26], and semantic/image segmentation [27–30]. These prior studies have validated the effectiveness of GANs as an image augmentation technique. The way to use a GAN-based augmentation model in the proposed method is similar to the ones in the literature. In other words, a collection of training data in the original dataset are utilized to train a GAN; then, the generator of the GAN can generate synthetic images that can be selectively added to the augmented dataset. The key difference between our work and the prior efforts is the proposed cTransGAN model, which inherits the merits of two powerful models, namely, cGAN [31] and TransGAN [32]. Experimental results on two datasets can demonstrate the superiority of the proposed method, compared to other GAN-based competitors in the area of image augmentation.

## 3. Materials and Methods

### 3.1. Dataset

We chose two datasets to validate the proposed method. The first one is a self-developed dataset for PCB component detection, and the second one is an existing dataset for PCB defect detection. We aim to verify that the proposed method can achieve superior performance on both tasks.

#### 3.1.1. Self-Developed Dataset

*(1) Dataset Basics*. Our dataset includes 2544 image samples that are divided into 3 categories: capacitors, diodes, and optocouplers. There are 1349 images of optocouplers, including 504 images for large optocouplers IC 1, 372 images for medium optocouplers IC100, and 473 images for small optocouplers. There are 799 images of capacitors, including 400 images for large capacitors and 399 images for small capacitors. There are 396 images of diodes. The basic information of the PCB dataset is illustrated in Table 1. The column “Original Dataset” explains the number of sample images for each category of electronic components. The next column “Portion in Original Dataset” explains the proportion of samples of each category in the original dataset. The next three columns provide the number of image samples of each category in the training set, the test set, and the generated dataset.

*(2) Dataset Acquisition*. Our PCB images are acquired using a BASLER camera, a2A5320-7gcBAS; an OPT lens, C1616-10M; and the light source by Haoli, HLFL478408K-K50. The height of both the lens and camera is 460 mm.

*(3) Dataset Preprocessing*. To alleviate complex calculation during training, we use the color image resolution fixed filling algorithm to process the images. The specific steps are as follows: the *X* and *Y* values (i.e., width and height) of the original image are obtained and compared with a predefined value (here 418); the image is then adjusted based on the comparison, where there are four cases to consider:

1. If *X* > 418 and *Y* <= 418, we use the NI visual function, IMAQ Resample, to change the *X* value of the image size to 418, and *Y* is fixed; then, we use the difference between *Y* and 418 and extend *Y* by ((418 − *Y*)/2) on both up and down directions and fill the pixels with zero.
2. If *X* <= 418 and *Y* <= 418, the same operations are performed on *Y* as in *A*; that is, we extend *Y* by ((418 − *Y*)/2) on both up and down directions and fill the pixels with 0. Then, the same operations are performed on *X*; that is, we extend *X* by ((418 − *X*)/2) on both left and right directions and fill the pixels with zero.
3. If *X* > 418 and *Y* > 418, the same NI visual function is used to change the *Y* value of the image to 418, and *X* is fixed; then, we use IMAQ resample to change the *X* value of the image size to 418, and *Y* is fixed.
4. If *X* <= 418 and *Y* > 418, the NI's visual function is performed to change the *Y* value of the image to 418, and *X* is fixed; then, we extend *X* by ((418 − *X*)/2) on both left and right directions and fill the pixels with zero.

**Figure 1:**
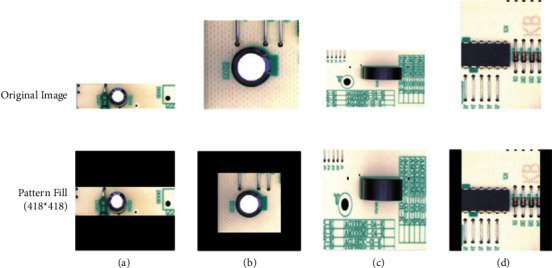
Illustration of color image resolution fixed filling algorithm for four cases (a–d).

**Figure 2:**
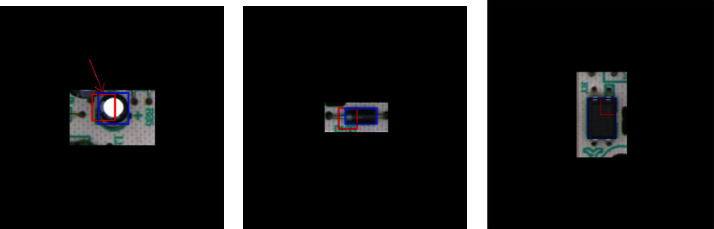
PCB samples. The red boxes marked in each image are for a different task and not used in this task. The blue boxes are the bounding box annotations used in this study. (a) Capacitor. (b) Diode. (c) Optocoupler.

The four cases are illustrated in Figure 1. Also, Figure 2 shows three samples, representing the three classes considered in this dataset.

#### 3.1.2. The DeepPCB Dataset

DeepPCB [33] is a dataset that contains 1,500 imagepairs, each of which includes a defect-free template image and an aligned testing image with annotations that include the positions of the six most common PCB defects: open, short, mousebite, spur, pin hole, and spurious copper.

All of the images in this dataset were captured using a linear scan CCD with a resolution of around 48 pixels per 1 millimetre. The defect-free template images are created by manually inspecting and cleaning sampled images in the manner described previously. The original size of the template and the image that was tested is approximately 16 k × 16 k pixels. Once this is done, the images are chopped into many smaller subimages of the same size as their parent image and aligned using template-matching techniques. Following that, a carefully chosen threshold is used to utilize binarization in order to avoid illumination disturbance. Although preprocessing algorithms can differ depending on the specific PCB defect detection algorithms used, the image registration and thresholding techniques used for high-accuracy PCB defect localization and classification are a common procedure used for PCB defect localization and classification.

**Figure 3:**
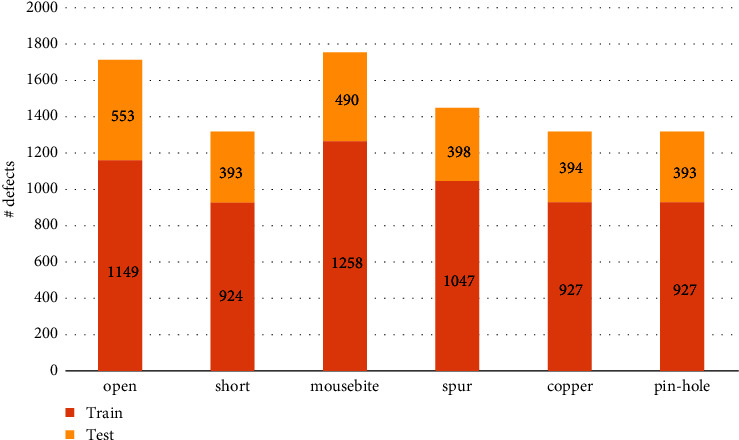
Number of defect classes in DeepPCB.

**Figure 4:**
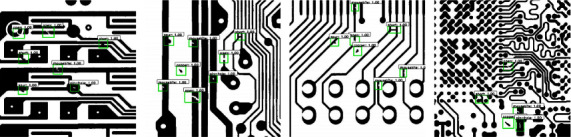
DeepPCB annotated samples.

Due to the fact that the real tested image has just a few defects, the authors augment the image by manually adding defects to each tested image in accordance with the PCB defect patterns, resulting in around 3 to 12 defects in each 640 × 640 image. After the annotation, the dataset is split into a training set with 1000 images and a test set with 500 images.  Figure 3 shows the number of the six defect classes for both training and test sets in DeepPCB. It is noted that the classes are relatively balanced in terms of quantity. We generated a total of 800 synthetic images with 600 instances for each defect class spread across the generated samples.  Figure 4 shows several annotated samples in DeepPCB.

### 3.2. System Overview

**Figure 5:**
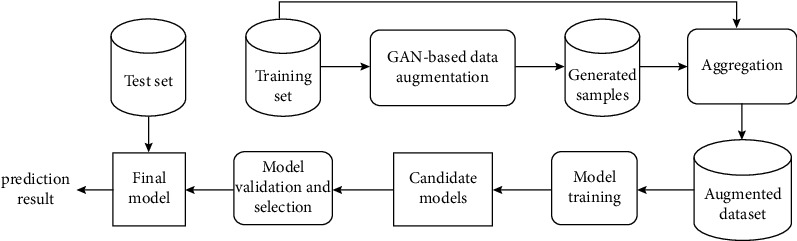
System architecture.

As shown in Figure 5, there are two stages in the workflow: data augmentation using TransGAN [32] and electronic component recognition using Faster R-CNN, YOLOv3, and SCNet [34]. TransGAN is an unsupervised deep learning method. Its generator is designed to be memory-friendly and consists of multiple stages, and each stage is formed by stacking several transformer encoders. In this paper, we use synthetic images generated by TransGAN and the annotations of the source images to augment the training dataset for Faster R-CNN, YOLOv3, and SCNet detectors.

### 3.3. Conditional TransGAN-Based Data Augmentation

In this subsection, we first provide a brief introduction of GAN, cGAN, and TransGAN, followed by a detailed description of the proposed cTransGAN.

#### 3.3.1. GANs

The vanilla GAN consists of two neural networks: a generator and a discriminator. The generator takes a random vector as input and attempts to create a synthetic data point that resembles a true sample from the original dataset. The discriminator, on the other hand, is trained with both real and fake samples and predicts whether a particular sample is real or not. To optimize the parameters, the prediction result is back-propagated via both networks.

cGAN improves on the vanilla GAN by conditioning the model on auxiliary information (e.g., class labels or *y*) to direct data production, allowing for more control over data modalities. Conditioning can be done by combining the generator and discriminator with a layer to generate a combined representation of *x* and *y*. The generator learns more semantic properties of a sample provided *y* after injecting *y*.

TransGAN [32] is an unsupervised deep learning method and uses a pure transformer with no convolution. Multiple transformer encoder blocks are utilized as building blocks for the discriminator and generator in TransGAN. A transformer encoder [35] is composed of a multihead self-attention component to obtain the long-term dependence between words in the sentence and the contextual semantic information. Even though the transformer was originally designed for natural language processing systems, it has been adopted in computer vision [36] areas. To mimic the sequential input required by the original transformer, the vision transformer (ViT) divides an input image into a collection of patches, which is the basis of TransGAN. Also, to reduce the memory cost caused by the numerous image patches, TransGAN develops a multistage memory optimization strategy to gradually increase/decrease the image resolution. Furthermore, TransGAN integrates a grid self-attention module, which converts an entire feature map to a grid of nonoverlapping feature patches. Next, it uses the local attention to replace the global attention in the grid, which greatly reduces the amount of calculation.

#### 3.3.2. cTransGAN

**Figure 6:**
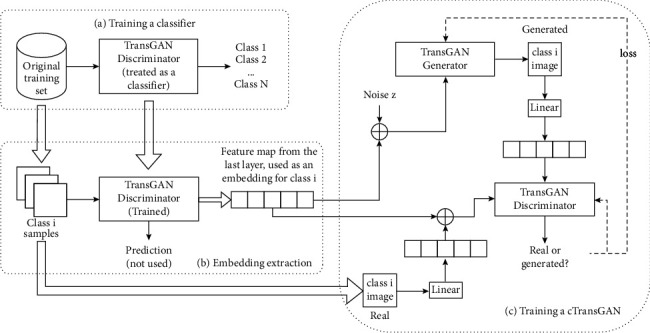
The architecture of cTransGAN.

Figure 6 shows the architecture of cTransGAN, which can be divided into three parts. First, we change the detection head of the TransGAN discriminator to output a tensor of size *N*, where *N* is the number of classes in the dataset. This way, the discriminator is treated as a classifier and trained using the original training set. The trained model is then used to produce an embedding for each class of samples. Specifically, the original training set is divided into multiple sets by class. We feed samples in class *i* into the trained model sequentially and extract the feature map from the last layer before the detection head and use it as the embedding, denoted by *e*_*i*_, to represent the class *i* samples. One major difference between the proposed method and cGAN is that we adopt the class embedding, rather than the label *y*, as an additional input of the GAN. The idea is inspired by the way how Word2Vec generates word embeddings. The strategy has been empirically effectively during training. After the embeddings are produced, they serve as inputs to guide the training of cTransGAN. As shown in Figure 6(c), the embedding for class *i* images, *e*_*i*_, is concatenated with a linearized real image of class *i*, and the result of concatenation is fed into the original TransGAN discriminator. Similarly, the concatenation of *e*_*i*_ and the noise vector *z* are fed into the original TransGAN generator, which aims to output a generated image of class *i*. Both the output representations of the cTransGAN discriminator and generator stay unchanged. Also, the internal neural structure and the optimization scheme remain unchanged.

Conditioned on the class embedding *e*_*i*_, the trained cTransGAN can generate high-quality synthetic images belonging to class *i*. The model can be trained end-to-end and speed up the process of data augmentation.

#### 3.3.3. Data Augmentation via GAN

A trained cTransGAN can be used to produce high-quality synthetic samples that look similar to the real samples. For the object detection task, the process involves the following steps. First, the marked bounding boxes in an annotated sample are warped, rescaled into the same size, and saved as images. This way, a collection of labeled images can be gathered to train a cTransGAN. After that, the trained cTransGAN generator can produce synthetic defect images that can be plugged back into the original sample image where the bounding boxes were warped from. Therefore, an augmented sample with synthetic defect annotations can be produced. For the task considered in DeepPCB, images belonging to the same class present clear patterns, which can be effectively captured by the cTransGAN.

### 3.4. Models

This subsection covers the models evaluated in this study for object detection.

#### 3.4.1. YOLOv3

**Figure 7:**
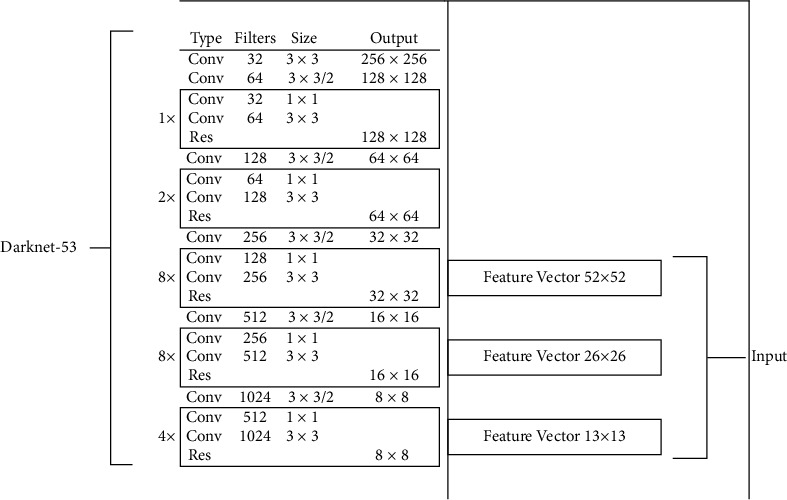
The neural architecture of Darknet-53.

YOLO (you only look once) is a fast object detection algorithm. As a good option for real-time detection without sacrificing too much accuracy, it can provide fast detection even though it is no longer the most accurate algorithm. It identifies a specific object in videos, images, or live feeds. YOLOv3 (YOLO version 3) [20] published in 2018, is the third improved version of YOLO, which is also the model we choose to use in this research. It uses Darknet-53 [9] instead of Darknet-19 [10] as its backbone network for feature extraction, inspired by SSD and ResNet [8]. The framework of Darknet-53 is shown in Figure 7. It is mainly composed of convolutional and residual structures. As illustrated in Figure 6, neither pooling layer nor fully connected layer is found in Darknet-53. Specifically, the last three layers: avgpool, connected, and softmax layer are used for classification training on the ImageNet dataset. The main components of Darknet-53 are 3 × 3 and 1 × 1 filters. It has 53 convolutional layers, more powerful and more efficient than the previous 19. There are residual connections, just as in ResNet. In forwarding propagation, changing the step size of the convolution kernel will change the size of the tensor. For example, Stride = 2 is corresponding to changing the length of the image to be half of it. YOLOv3 splits the image into seconds × small cells. Each grid unit predicts three components: (1) the coordinates of the B bounding box (*x*, *y*, *w*, *h*), (2) the confidence score P (object), and (3) C conditional class probability, which is conditional based on the presence of an object in the grid cell. YOLOv3 makes predictions over three different scales and uses nine anchor boxes, three for each scale. There could be a loss of precision in small structures because of low-resolution 3D methods. In this research, YOLOv3 is trained with 450 epochs and with a decreasing learning rate.

#### 3.4.2. SCNet

SCNet can establish a convolutional neural network structure of semantic correspondence between images of different instances of the same object or scene category. It is used to learn geometrically plausible models for semantic correspondence. In SCNet, regional proposals are used as matching primitives, and geometric consistency is explicitly added to its loss function. Image pairs obtained from the PASCAL VOC 2007 keypoint dataset are used to train SCNet. In this research, we use ResNet101 [37] as the backbone of SCNet for training. ResNet101 [37] is residual network with 101 layers. Each layer is composed of an identity block and convolution block. Also, the skip connections in ResNet allow alternate shortcut for the gradient to flow through. It also allows the model to learn identity functions which make sure the higher layer will perform as good as or better than the lower layer, but not worse.

#### 3.4.3. Faster R-CNN

R-CNN is the pioneering work of two-stage algorithms for object detection. There are mainly three modules in R-CNN: region proposal, vector transformation, and classification. SSP-net [38] improves R-CNN in multiple areas including detection performance. Fast R-CNN [39] is a combination of R-CNN and SSP-net. Compared with various CNN technologies, the main advantage of Fast R-CNN is that it uses selective search to generate region proposals, which greatly saves time and improves the accuracy of object detection. Faster R-CNN uses the regional proposal network (RPN) to replace the selective search module in Fast R-CNN, which further improves the time efficiency and accuracy of object detection.

The regional proposal network (RPN) uses a fully convolutional network to generate rectangular object proposals from the dataset images. It uses the input image to create anchor points or region boxes and predicts whether an anchor is in the background or the foreground. It then selects the area frame with the most region proposals as the optimal proposal. It improves the efficiency of regional proposals and accurately detects objects. The task is to mark the anchor with the highest overlap with the ground truth box as the foreground and the anchor with the lowest overlap as the history. Therefore, each anchor is considered to be a foreground or background with a predicted label. After RPN, a region proposal can be obtained with feature maps of various sizes. However, it is difficult to process feature maps of different sizes.

**Figure 8:**
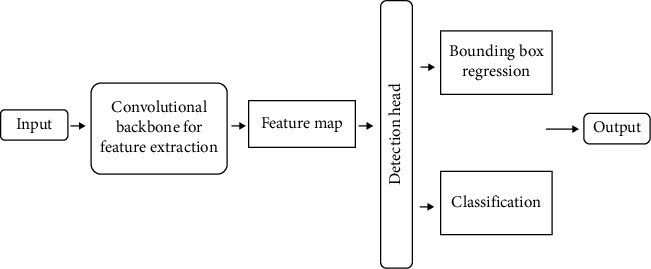
The framework of Faster R-CNN.

Faster R-CNN uses a region proposal network faster than R-CNN. In R-CNN, pixel-level region proposals are used as input while in Faster R-CNN, feature map-level region proposals are taken as input. As explained in [37], using the combination of Faster R-CNN and ResNet101 can improve the performance of the network. The framework of Faster R-CNN is illustrated in Figure 8.

## 4. Experiments and Results

### 4.1. Experiment Settings

We conduct experiments on a computer with Windows 10, which is equipped with 16 GB RAM and Intel Core i7-8700 CPU. The TensorFlow framework and Nvidia GeForce RTX 2070 GPU are used to train the DCNN model. The program uses Python 3.6.7.

### 4.2. Model Training

To train TransGAN, we use the Adam optimizer, a batch size of 64 for both the discriminator and the generator, and a learning rate of 0.0001. The cTransGAN model has been trained for 240 epochs. We train Faster R-CNN ResNet101, YOLO V3 DarkNet-53, and SCNet ResNet101 models on the original training set and the augmented training set. The three deep learning models combined before or after using data augmentation, and there are 12 models in total for comparative study, as shown in Table 2 in Subsection 4.4. An image is taken as the input for each training model that can detect the bounding boxes of the detected PCB objects within the image. Each bounding box consists of the predicted category and confidence. The chosen hyperparameters include a momentum of 0.7, a verification period of 4000, a learning rate of 0.004, a weight decay of 0.0004, a batch size of 32. The training was conducted for 2000 epochs.

### 4.3. Evaluation Metrics

The mean average precision (mAP) is the primary performance metric, which is also a commonly used performance indicator in object detection. In addition, we consider four classification metrics, including accuracy (Acc), recall (Rec), specificity (Spe), and *F*1-score (F1). The mAP is calculated based on Intersection overUnion (IoU). IoU is a metric to evaluate how similar our predicted bounding box is to the ground truth bounding box. It is the ratio of the intersection area and the combined area between the prediction and the ground truth bounding box. Normally, if IoU > 0.5, it is a true positive (TP); otherwise, it is a false positive (FP). Furthermore, if (1) no detection at all or (2) detection of IoU > 0.5 but the object being misclassified, it is a false negative (FN). Precision (Pre) is the ratio of true positives (TP) to the total number of predicted positives. Recall (Rec) is the ratio of TP to the total number of ground truth positives. The interpolation precision is calculated at each recall level by taking the maximum precision of that level. Then, we calculate the average precision (AP) by taking the precision and checking the area under the curve. The mAP is the mean value of AP calculated for all categories of all images in the test set. Generally speaking, the higher the mAP value, the better the model. The definitions of these four indicators are as follows:

(1) $$\begin{array}{c} Acc=\frac{TP+TN}{TP+TN+FP+FN}\text{,}\\ Spe=\frac{TN}{TN+FB}\text{,}\\ Rec=\frac{TP}{TP+FN}\text{,}\\ F1=\frac{Pre\times Rec}{Pre+Rec}\text{.} \end{array}$$

### 4.4. Comparative Results

We evaluated three object detection models, including Faster R-CNN ResNet101, YOLOv3 DarkNet, and SCNet ResNet101, on both datasets. Each model was validated with four settings, including without augmentation, with image processing-based augmentation (referred to as IPAug), with TransGAN, with cGAN, and with cTransGAN. For IPAug, we defined a set of transformation algorithms including blur, flip, center crop, CLAHE, color jitter, rotate, and transpose. For each image to be augmented, we randomly selected three transformation algorithms applied to the image to generate an augmented image. Therefore, these experiments also serve as an ablation study. We provide the result interpretation, insights, and analysis as follows.

*(1) Results on the Self-Developed Dataset*. Table 2 shows the results of the three models under four experimental settings in mAP. We have listed mAP for each object class as well as the overall mAP, i.e., the last column. The highest score for each column is marked in bold. We have the following findings.

- IPAug was slightly worse than the GAN-based augmentation methods for all three models. However, the difference between IPAug and TransGAN was minor, with a gap less than 1%.
- The adopted four settings for each model form an ablation study that evaluates the effect of each added module. Since cTransGAN can be regarded a combination of cGAN and TransGAN, it is expected to inherit the merits of both GANs. Our results can confirm this hypothesis. It is observed that if the training set is augmented by either TransGAN or cGAN, the mAP improves by 0.7%–1.3% across all three models, compared to base setting where augmentation is not applied. Moreover, when augmented by cTransGAN, the overall mAPs have been further lifted by 0.7%–1.0%. The mAP gains for each individual class have also been consistent for all models.
- The best performing model out of the three was SCNet RestNet101 augmented by cTransGAN with an mAP of 96.2%, outperforming Faster R-CNN ResNet101 and YOLOv3 DarkNet by 2.8% and 1.6%, respectively.
- These results demonstrate the effectiveness of cTransGAN in generating high-quality synthetic images to enhance the diversity and quantity of the dataset used for training.

*(2) Results on the DeepPCB Dataset*. The authors of [33] have provided a strong baseline model, which is a custom deep neural network that utilizes a group pyramid pooling (GPP) technique to obtain features of different resolutions from a pyramid pooling structure. The pooling operation can be either average or max pooling. Their results showed that GPP with max pooling presented the best score. Thus, we consider GPP with max pooling as the SOTA on this dataset. The results are shown in Table 3, where the first three sections cover the results of the three models used in this study, and the last section shows the SOTA. Similar to the self-developed dataset, each model has been validated with four settings. We provide the observations as follows.

- Similar observations were found for IPAug, which presented comparable scores with TransGAN but was worse than cGAN and cTransGAN. Our experiments can validate that GAN-based augmentation methods are generally superior to IPAug.
- Despite the differences in task characteristics and object patterns between the two datasets, the GAN-based data augmentation strategy has consistently improved the mAP for both tasks. In the DeepPCB dataset, the gains brought by cGAN and TransGAN are in the range of 0.1% and 1.2%. The low end, i.e., a gain of 0.1% is observed for the Faster R-CNN ResNet101 model with TransGAN. Given that the mAP for the base setting of Faster R-CNN ResNet101 has been relatively high (97.7%), the effect of TransGAN became limited. However, the gains brought by the proposed cTransGAN have been impressive, in the range of 1.1% (for Faster R-CNN ResNet101) and 2.7% (for YOLOV3 DarkNet).
- Our best model, Faster R-CNN ResNet101 with cTransGAN has an mAP of 98.8%, posting an 0.1%gain compared to the SOTA. Despite the minor gain, the results show that a comparable performance can be obtained via cTransGAN-based data augmentation applied to an existing model. The point we made is that the efforts on data, rather than model, are as effective.

## 5. Conclusion

PCB is prone to open circuit, short circuit, or magnetic leakage during manufacturing. In order to automate the identification of PCB electronic components, we have established an image dataset that includes three categories of PCB components, namely optocouplers, capacitors, and diodes. In addition, we propose cTransGAN to generate synthetic samples, which effectively enhance the scale and diversity of the original training set. Three deep learning models, including Faster R-CNN ResNet101, YOLO V3 DarkNet-53, and SCNet ResNet101, are trained and evaluated on the datasets. We also design plenty of comparative experiments to verify the effectiveness of object detection. The results have demonstrated that the augmentation method based on cTransGAN makes the image samples more diversified, so that the models can capture better semantic features, thereby obtaining significant performance improvement. Based on the experimental results, with data augmentation using cTransGAN, SCNet ResNet101 achieves the best detection accuracy. In addition to the self-developed dataset, we also evaluated cTransGAN on DeepPCB, a dataset for PCB defect detection, and similar observations can be found as well. In summary, the superiority of cTransGAN has been validated on two datasets to handle two different PCB object detection tasks.

Meanwhile, there are some limitations in this study, which will be addressed in future work. First, classic image processing-based augmentation can be used together with cTransGAN-based data augmentation to quickly obtain larger number and greater diversity of datasets. It will be interesting to explore the individual and combined effects of these two types of augmentation, and how they complement each other to maximize the benefits of augmentation. Second, this paper only studies cTransGAN architecture, because its performance in generating high-quality synthetic images has been verified. As a future work, many GAN options can be studied. Third, the samples generated by the GAN-based augmentation need to be manually selected and labeled before they can be used for training, which is very time-consuming. It is worthwhile to develop additional supporting algorithms to facilitate the application of the generated samples.

## Figures and Tables

**Table 1:** PCB dataset.

	Original dataset	Percentage (%)	Training set	Test set	Generated images
Optocoupler	1349	50.03	944	405	1200
Diode	396	15.57	277	119	400
Capacitor	799	31.41	559	240	800
Total	2544	100.00	1780	164	1200

**Table 2:** Experimental results (mAP in percentage) on the self-developed dataset. The highest scores for each column are marked in bold.

Models	Settings	Capacitor	Diode	Optocoupler	mAP
**Faster R-CNN ResNet101**	w/o augmentation	90.1	91.8	92.6	91.5
	W/IP aug	90.4	92.5	93.3	92.1
	w/TransGAN	90.7	93.2	93.0	92.3
	w/cGAN	91.2	92.9	93.1	92.4
	w/cTransGAN	93.0	93.9	93.5	93.4

**YOLOV3 DarkNet**	w/o augmentation	92.6	93.1	94.2	93.3
	w/IP aug	93.3	93.1	94.8	93.7
	w/TransGAN	93.1	93.2	95.6	94.0
	w/CGAN	94.0	94.5	95.2	94.6
	w/cTransGAN	94.9	95.1	95.8	95.3

**SCNet ResNet101**	w/o augmentation	93.8	94.1	94.5	94.1
	w/IP aug	94.5	94.7	96.3	95.2
	w/TransGAN	94.7	95.3	95.9	95.3
	w/cGAN	94.1	95.4	96.7	95.4
	w/cTransGAN	**95.2**	**95.5**	**97.9**	**96.2**

Table 2 gives the metrics for the best models trained by the Faster R-CNN ResNet101, YOLOV3 DarkNet, and SCNet ResNet101 models using different enhancements on the two dataset tasks, respectively, where the metrics include the AP value and the mean AP value (mAP) for each subtarget. The range of AP values is from 0% to 100%, with higher values demonstrating better detection of the target. As can be seen in Tables 2, the detection results using cTransGAN are almost always optimal.

**Table 3:** Experimental results (mAP) on the DeepPCB dataset.

Models	Settings	Open	Short	Mousebite	Spur	Copper	Pin-hole	mAP
**Faster R-CNN ResNet101**	w/o augmentation	94.8	95.7	98.5	98.8	98.9	99.5	97.7
	W/IPAug	95.6	96.3	98.2	98.7	98.8	99.4	97.8
	w/TransGAN	95.4	96.1	97.9	98.4	99.1	**99.6**	97.8
	w/CGAN	96.2	97.6	98.2	98.3	98.9	99.5	98.1
	w/cTransGAN	98.1	98.3	**99.2**	**98.9**	**99.1**	99.2	**98.8**

**YOLOV3 DarkNet**	w/o augmentation	91.2	92.4	94.8	92.3	96.4	93.2	93.4
	W/IPAug	92.4	93.2	94.6	92.5	96.8	93.7	93.9
	w/TransGAN	92.8	93.6	94.9	92.4	97.1	94.5	94.2
	w/cGAN	93.5	93.5	94.7	92.6	97.8	94.1	94.4
	w/cTransGAN	95.2	95.6	96.1	94.3	98.1	97.2	96.1

**SCNet ResNet101**	w/o augmentation	93.6	93.8	96.2	96.9	97.5	99.2	96.2
	W/IPAug	94.5	95.2	98.1	97.3	97.9	99.1	97.1
	w/TransGAN	94.7	95.9	97.6	96.9	97.9	99.2	97.0
	w/CGAN	95.1	96.4	98.4	97.2	97.8	99.3	97.4
	w/cTransGAN	97.5	96.9	98.9	98.1	98.4	99.3	98.2

**SOTA** [33]	w/max pooling	**98.5**	**98.5**	99.1	98.2	98.5	99.4	98.7

Table 3 gives the metrics for the best models trained by the Faster R-CNN ResNet101, YOLOV3 DarkNet, and SCNet ResNet101 models using different enhancements on the two dataset tasks, respectively, where the metrics include the AP value and the mean AP value (mAP) for each subtarget. The range of AP values is from 0% to 100%, with higher values demonstrating better detection of the target. As can be seen in Tables 3, the detection results using cTransGAN are almost always optimal.

## Data Availability

The data used to support the findings of this study are available at https://github.com/long-deep/pcb-detect and https://github.com/tangsanli5201/DeepPCB.
